# Digital Dental Radiology and Diagnostics—From 2D to 3D

**DOI:** 10.1111/adj.70024

**Published:** 2025-11-24

**Authors:** Matheus L. Oliveira

**Affiliations:** ^1^ Department of Oral Diagnosis Piracicaba Dental School, Universidade Estadual de Campinas Piracicaba SP Brazil

**Keywords:** artificial intelligence, cone‐beam computed tomography, diagnostic imaging, diffusion of innovation, technology

## Abstract

Digital dental radiology has evolved significantly from traditional two‐dimensional (2D) imaging to advanced three‐dimensional (3D) modalities such as cone‐beam computed tomography (CBCT). This progression has overcome many limitations of 2D imaging, including superimposition and distortion, enabling more accurate visualisation of complex anatomical structures. Despite CBCT's higher radiation dose compared to 2D imaging, ongoing advances in low‐dose protocols and artefact reduction algorithms have expanded its clinical applications. Integration with digital tools such as intraoral scanners and CAD/CAM systems has further enhanced its utility in guided implant surgery, orthodontic appliance design, and forensic dentistry. Emerging artificial intelligence technologies promise to improve image analysis, diagnostic accuracy, and workflow efficiency. Emphasising radiation safety and careful imaging selection remains essential. Additionally, magnetic resonance imaging and ultrasound are gaining attention as non‐ionising imaging alternatives, offering valuable soft tissue assessment complementary to CBCT's strengths in hard tissue evaluation. Together, these innovations reflect the crucial change in dental diagnostics from 2D to 3D imaging, advancing patient care through improved accuracy and comprehensive treatment planning.

## Clinical Relevance Statement

The evolution from 2D to 3D imaging has fundamentally changed dental diagnostics, providing clinicians with enhanced visualisation and improved diagnostic confidence. This review critically examines how technological progress, from digital radiography to cone‐beam computed tomography and emerging non‐ionising modalities, has influenced clinical decision‐making, treatment precision, and patient safety. By synthesising current evidence and practical considerations, it offers clinicians guidance on selecting appropriate imaging modalities to balance diagnostic benefit, radiation exposure, and cost‐effectiveness in everyday dental practice.

## 1 Introduction

Over the past few decades, dental radiology has undergone a remarkable transformation, evolving from conventional film‐based imaging to sophisticated digital technologies that have reshaped diagnostic capabilities and clinical workflows. The initial transition to digital two‐dimensional (2D) radiography brought significant improvements in image quality, efficiency, and radiation dose reduction, establishing new standards in diagnostic precision. However, the inherent limitations of 2D imaging, particularly the superimposition of anatomical structures and the inability to accurately depict complex spatial relationships, have driven the development and adoption of three‐dimensional (3D) imaging modalities.

Cone‐beam computed tomography (CBCT) has emerged as an essential tool in dental diagnostics, offering enhanced visualisation of hard tissues and enabling more precise treatment planning across various specialties, including implantology, endodontics, orthodontics, and oral surgery. Moreover, the increasing reliance on ionising radiation–based modalities has also raised important considerations regarding radiation exposure and patient safety.

This article provides a critical overview of the evolution from 2D to 3D imaging in dental diagnostics, examining the strengths, limitations, and clinical applications of current technologies. Furthermore, it explores emerging trends including the integration of artificial intelligence (AI) into image acquisition and interpretation, the evolution of digital workflows and the future integration of non‐ionising imaging modalities, such as magnetic resonance imaging (MRI) and ultrasound, that hold the promise of further advancing dental diagnostics while minimising radiation risks.

## 2 The Era of Digital 2D Imaging

The transition from conventional film‐based radiography to digital 2D imaging has revolutionised dental diagnostics and practice management. Digital intraoral radiography, panoramic imaging, and cephalometric radiography are fundamental components of routine dental care, offering numerous advantages over their analog precursors; yet, this cannot be assumed to reflect the current global reality, as significant disparities in access to digital technologies persist. These inequalities highlight that the economic value of digital radiography is highly context‐dependent, thus affecting cost‐utility, and that investments in technology may not yield equivalent benefits across different healthcare settings.

The shift from film to digital imaging receptor, including charge‐coupled device, complementary metal‐oxide‐semiconductor, and photon‐counting sensors, and photostimulable phosphor plates, has substantially improved image acquisition speed, patient experience, and diagnostic accuracy. A recently published study identified a total of 150 intraoral digital radiographic imaging systems, including both historical and current models, of which 70% were sensor‐based and 30% were photostimulable phosphor plate‐based systems [1, 84]. Digital systems allow for rapid image visualisation, eliminating the need for chemical processing and reducing the environmental impact associated with traditional film development. Clinically, digital intraoral radiographs provide excellent spatial resolution for the detection of caries, periodontal bone loss, and periapical pathology [2, 3, 4]. Image enhancement tools, such as brightness and contrast adjustments, and magnification further support diagnostic decision‐making. Importantly, digital receptors are more sensitive to X‐rays than film and have a greater dynamic range, allowing for lower radiation doses without compromising image quality [5, 6]. However, the 2D nature of intraoral radiographs inherently limits their diagnostic utility by superimposing anatomical structures and potentially obscuring key pathological findings.

**FIGURE 1:**
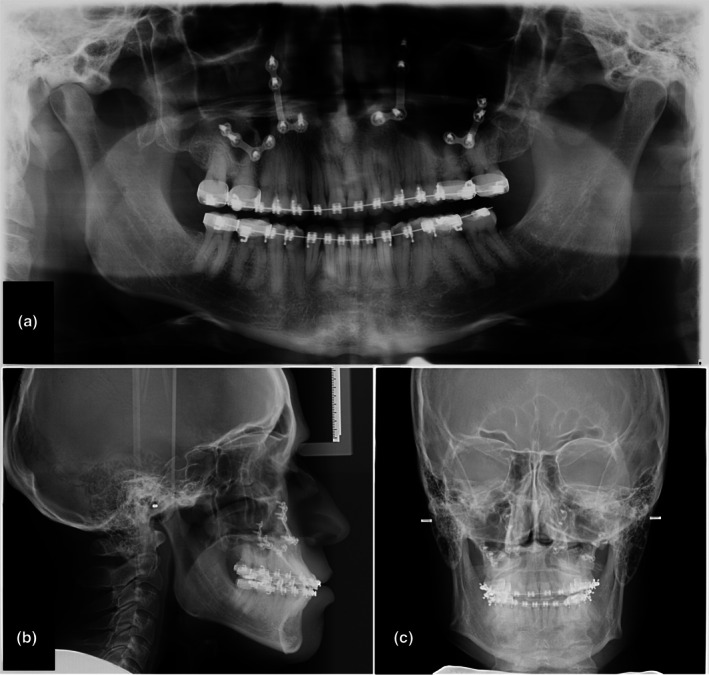
Postoperative digital radiographic assessments of the same patient following orthognathic surgery: (a) panoramic radiograph, (b) lateral cephalometric projection, and (c) posteroanterior (frontal) cephalometric projection.

Panoramic radiography offers a broad overview of the maxillofacial structures, including the dentition, jaws, temporomandibular joints, and adjacent anatomical landmarks. The digitization of panoramic systems has enhanced image clarity, reduced radiation exposure, and improved workflow efficiency (Figure 1a). Modern panoramic units incorporate advanced positioning aids and exposure protocols to minimise patient mispositioning and optimise image quality. Despite these advancements, panoramic radiographs maintain several limitations intrinsic to their design. The technique involves complex rotational movements that may introduce geometric distortions, magnification errors, and the generation of ghost images. Additionally, overlapping structures and lower spatial resolution compared to intraoral radiographs can hinder the detection of subtle lesions or fine anatomical details [7]. Therefore, panoramic imaging serves best as a screening tool rather than a definitive diagnostic modality.

Cephalometric radiography remains indispensable in orthodontic diagnosis and treatment planning, offering standardised lateral and posteroanterior views of craniofacial structures. The transition to digital cephalometry has streamlined image acquisition and analysis, facilitating automated landmark identification, growth assessment, and simulation of orthodontic interventions (Figure 1b,c). Furthermore, extraoral bitewing and occlusal radiographs, enabled by digital systems, provide valuable diagnostic alternatives in cases where intraoral imaging is contraindicated [8]. Nevertheless, 2D imaging is inherently limited in its representation of three‐dimensional structures. Superimposition, distortion, and the inability to assess the spatial relationships of anatomical features restrict the diagnostic precision of 2D modalities, particularly in complex cases involving impacted teeth, root fractures, or maxillofacial trauma.

Alongside technological advancements in digital 2D imaging, AI has emerged as a transformative tool in dental radiology [9]. AI‐driven analysis of intraoral and extraoral radiographs, including bitewing, periapical, and panoramic images, has gained significant attention, with numerous studies demonstrating automated detection of caries [10], periodontal bone loss [11], periapical lesions [12], and other pathologies. Deep learning algorithms, particularly convolutional neural networks, have shown promising accuracy and reproducibility, potentially supporting clinical decision making, reducing diagnostic variability, and enhancing workflow efficiency [13]. While much of this research is still in the validation phase, AI applications in 2D imaging represent a rapidly evolving area of investigation, highlighting the growing integration of computational tools into routine dental diagnostics. However, the adoption of these technologies requires substantial investment in computational infrastructure and in the proper training of AI models, which may limit their accessibility and overall value in certain clinical settings.

## 3 The Advent of 3D Imaging in Dentistry

The advent of CBCT has fundamentally transformed dental diagnostics by introducing 3D imaging into routine practice. Unlike 2D modalities that project complex anatomical structures onto a flat plane, CBCT allows for detailed spatial visualisation, enabling clinicians to assess intricate anatomical relationships with precision. Since its introduction in the late 1990s, CBCT has gained widespread acceptance across multiple dental specialties, driven by technological advances that optimise image quality, reduce radiation exposure, and improve accessibility.

### 3.1 Technical Principles of CBCT

CBCT, which formerly employed a cone‐shaped X‐ray beam, now uses a pyramidal‐shaped beam and a square‐shaped flat‐panel detector that rotates around the patient, capturing multiple sequential projections in a single scan. These data are subsequently reconstructed using specialised algorithms to generate volumetric datasets composed of isotropic voxels, i.e., voxels with equal dimensions in all directions. This isotropic nature ensures equal resolution in all spatial dimensions, enabling accurate multiplanar reconstructions (axial, sagittal, and coronal) and the creation of 3D renderings [14, 15].

**FIGURE 2:**
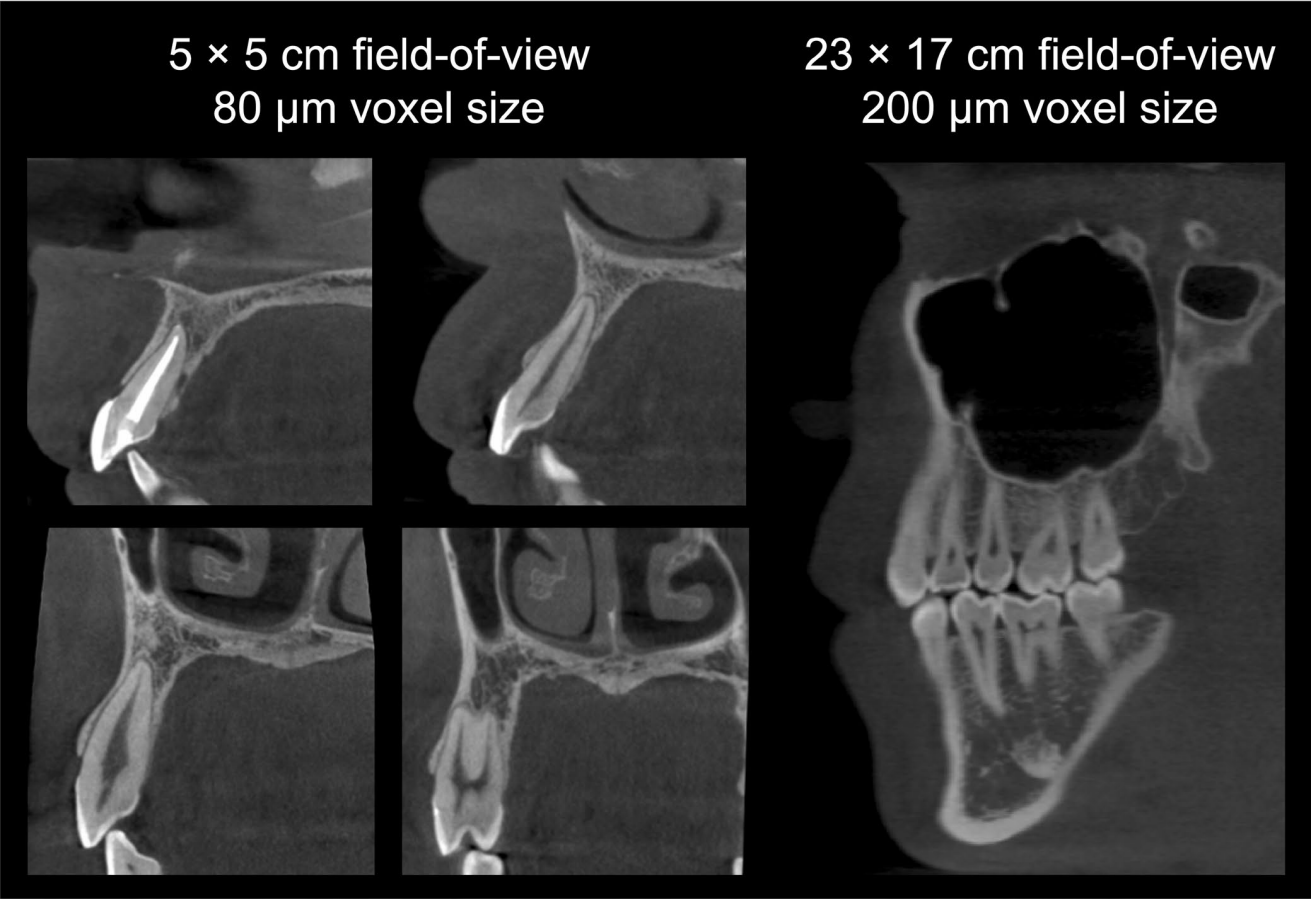
Cropped cross‐sectional cone‐beam computed tomography images obtained at two different field of view and voxel resolutions. Note the difference in overall sharpness when evaluating, for example, the periodontal ligament space and trabecular bone.

One of CBCT's significant advantages over multi‐slice computed tomography (MSCT), also referred to as medical CT, lies not only in its relatively low radiation dose, mainly achieved through reduced tube current, but also in its higher spatial resolution [16]. Additionally, CBCT machines are typically more compact, cost‐effective, and better suited to the dental operatory environment than traditional CT scanners. Nevertheless, CBCT involves a balance between image resolution and field of view (FOV). Small FOV scans offer high‐resolution imaging ideal for endodontic applications, whereas large FOV scans provide broader anatomical coverage at the expense of detail (Figure 2). Therefore, selecting an appropriate FOV tailored to the specific diagnostic task is essential to optimise image quality and minimise radiation exposure [9, 17, 18]. The clinical utility of CBCT includes a wide range of dental and maxillofacial applications, enhancing diagnostic accuracy, treatment planning, and patient outcomes.

### 3.2 Clinical Applications of CBCT

**FIGURE 3:**
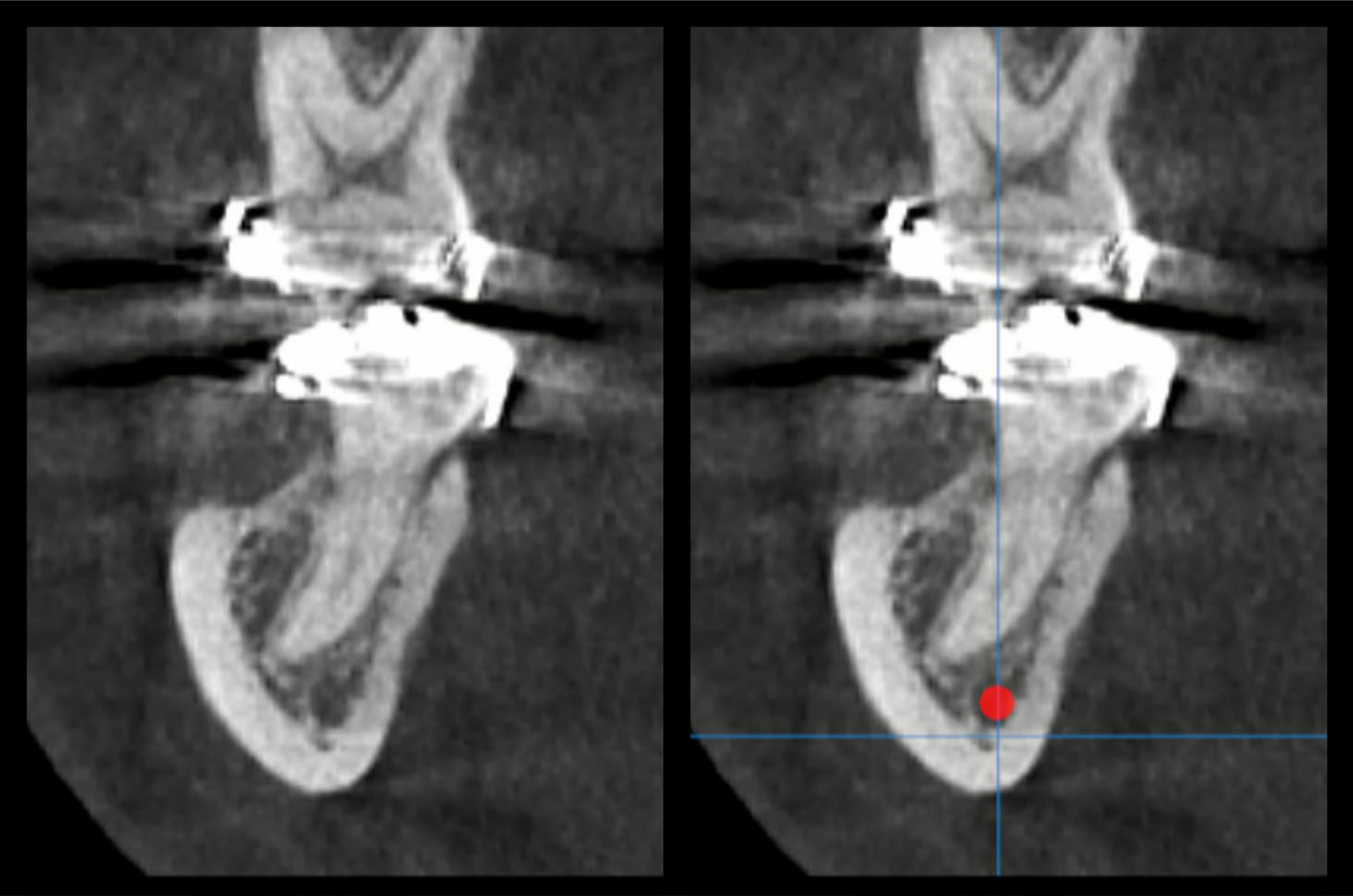
Cross‐sectional cone‐beam computed tomography image of the posterior mandibular region, highlighting the spatial relationship between the root apex of a molar and the mandibular canal (solid red circle).

**FIGURE 4:**
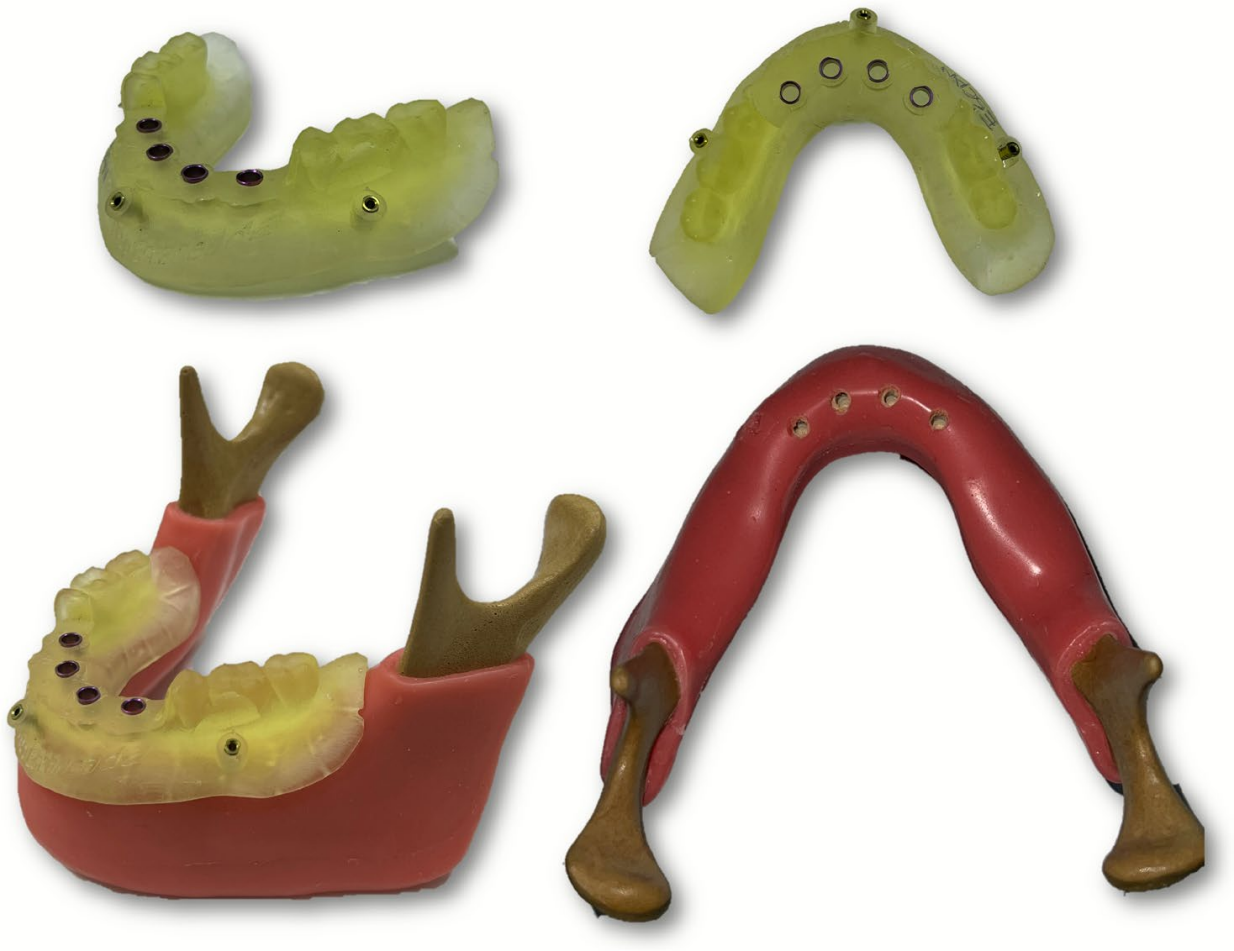
Photographs of a computer‐aided design and manufacturing surgical guide alone and positioned on a synthetic mandible to accurately guide implant perforations.

**FIGURE 5:**
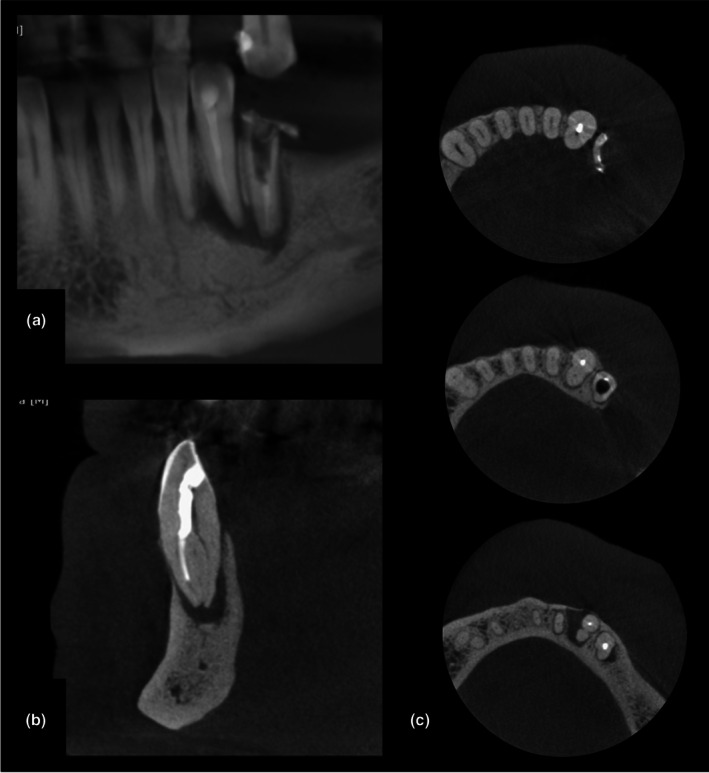
Lower canine presenting an untreated lingual radicular canal in cone‐beam computed tomography images: (a) 10‐mm‐thick panoramic reconstruction; (b) cross‐sectional image of the canine; (c) axial reconstructions at three levels from cervical to apex.

CBCT has become indispensable in multiple dental fields, as volumetric imaging allows for the identification of critical anatomical structures such as the inferior alveolar nerve, maxillary sinus, and adjacent teeth, facilitating risk mitigation and optimal implant placement (Figure 3). Furthermore, CBCT datasets can be integrated with computer‐aided design and manufacturing (CAD/CAM) technologies to fabricate surgical guides, ensuring accuracy during implant placement procedures (Figure 4). In implant dentistry, CBCT enables precise assessment of alveolar bone dimensions, morphology, and quality, supporting guided implant placement [19, 20, 21, 83]. However, there is no current evidence to support the use of CBCT as a standard postoperative procedure to evaluate peri‐implant bone mainly due to the formation of metal‐related artefacts [22, 23]. In endodontics, CBCT provides enhanced visualisation of root canal anatomy, periapical lesions, root fractures, and resorptive defects that may be undetectable or obscured on conventional 2D radiographs [2, 24, 25]. The ability to assess teeth in multiple planes reduces diagnostic uncertainty, particularly in complex cases involving teeth with intricate canal configurations or atypical pathology (Figure 5). Additionally, CBCT contributes to the diagnosis of periodontal diseases, especially in pre‐surgical treatment planning for complex periodontal defects, enabling precise assessment of bone morphology, bone defects, and the spatial relationships between teeth and surrounding structures [26, 27, 28].

**FIGURE 6:**
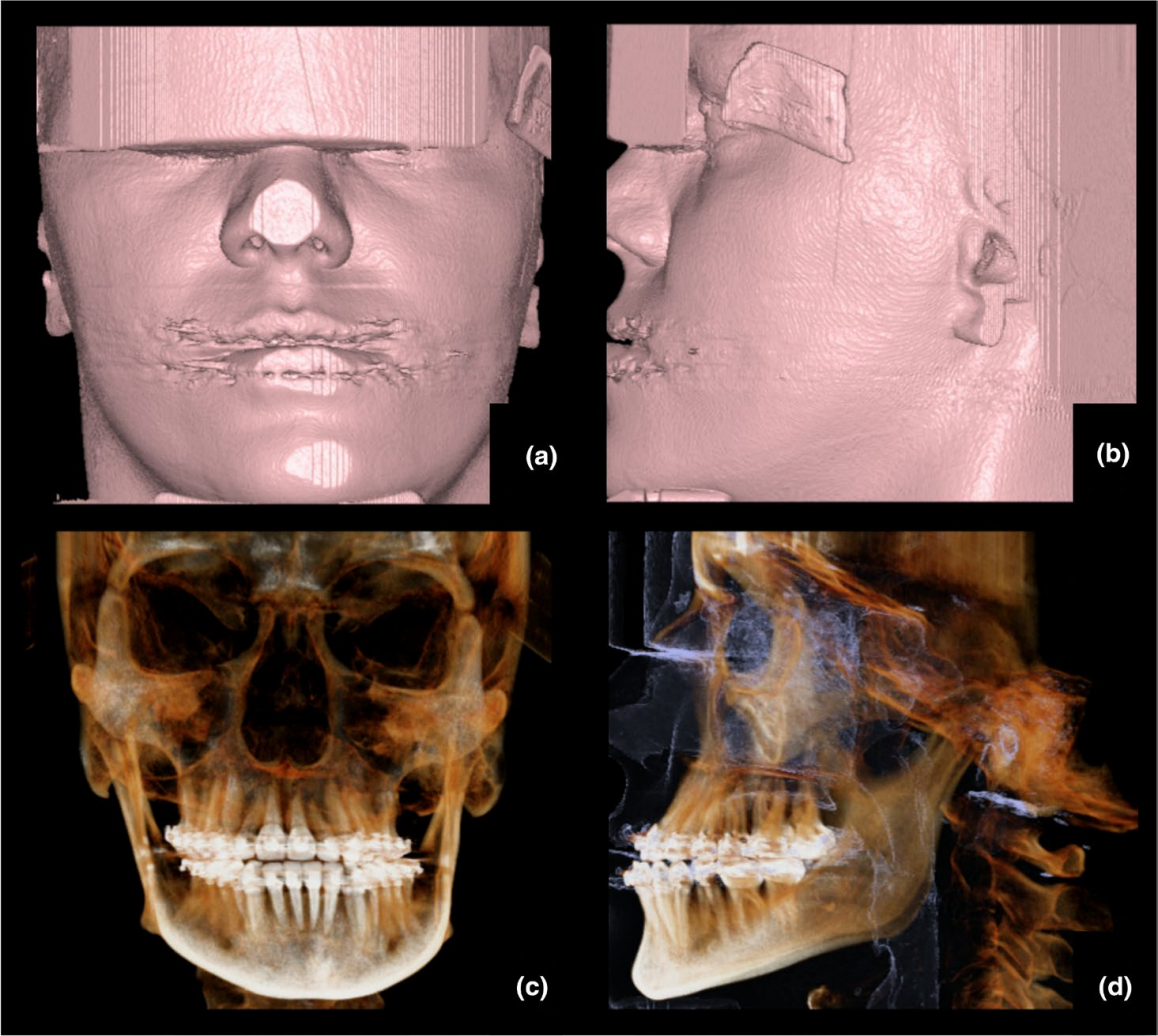
Cone‐beam computed tomography volume renderings. Frontal (a, c) and lateral (b, d) views of the soft tissue surface (a, b), bone (c), and bone with airways (D).

Applications of CBCT in orthodontics include the evaluation of impacted teeth, trauma, cleft lip and/or palate, congenital anomalies and syndromes, and temporomandibular joint (TMJ) morphology [17, 29, 30]. The availability of 3D cephalometric analysis has further refined orthodontic diagnosis and treatment planning in complex cases (Figure 6), although concerns regarding radiation dose necessitate careful use, particularly in paediatric populations. CBCT offers high‐resolution imaging of osseous components of the TMJ, enabling the diagnosis of degenerative changes, fractures, and developmental anomalies. However, its inability to adequately visualise soft tissues such as the articular disc limits its utility for comprehensive TMJ evaluation, necessitating complementary modalities such as MRI in certain cases.

**FIGURE 7:**
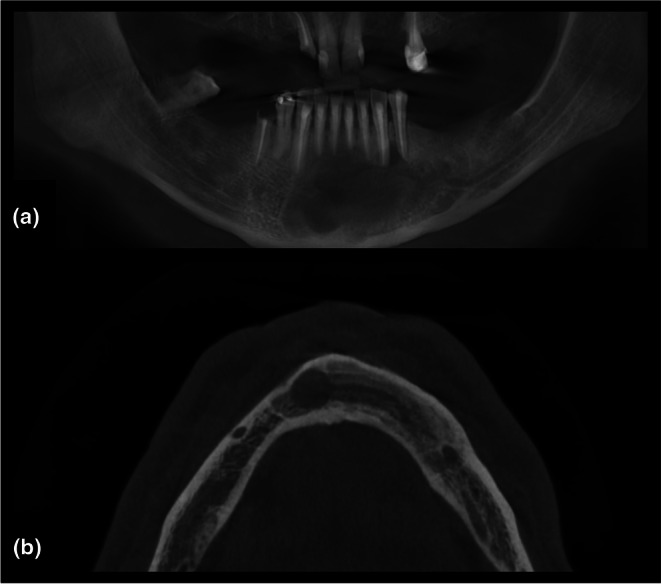
Cropped cone‐beam computed tomography images revealing a radiolucent lesion in the anterior mandible: (a) 15‐mm‐thick panoramic reconstruction; (b) axial reconstruction. Note the absence of buccolingual expansion, which is more clearly appreciable in the axial view, underscoring its added diagnostic value.

CBCT's capacity to delineate the extent of pathological lesions—including cysts, tumours, and osteomyelitis—as well as to assess maxillofacial fractures and dental trauma, has made it a vital diagnostic tool in oral and maxillofacial surgery [31]. The spatial information provided by 3D imaging facilitates surgical planning and aids in prognostic evaluation (Figure 7).

### 3.3 Strengths and Limitations of CBCT

CBCT overcomes the limitations of superimposition and distortion inherent in 2D imaging, allowing for accurate representation of complex anatomical structures. The availability of multiplanar and 3D reconstructions enables precise localization and characterisation of lesions and anatomical variations, leading to more informed clinical decisions. Furthermore, CBCT data can be seamlessly integrated with intraoral scans, CAD/CAM systems, and 3D printing technologies, supporting advanced treatment modalities such as guided implant surgery and custom prosthetics [32].

Despite these advantages, CBCT does have limitations. Although it generally delivers lower radiation doses than medical CT, the dose typically remains higher than that of standard 2D dental radiography [7, 33]. Therefore, its use must be justified based on clinical need, following the principles of radiation protection. Excessively detailed CBCT scans aimed at producing “pretty images”, when a diagnostically adequate image would suffice, expose patients to unnecessary radiation and compromise their safety. In addition, the considerable variability in exposure parameters across different CBCT systems renders dose quantification challenging, underscoring the necessity for meticulous, case‐specific justification [33]. Additionally, the presence of metallic restorations, implants, and orthodontic appliances can generate artefacts such as beam hardening and scatter, potentially degrading image quality and complicating interpretation [23, 34, 35, 36]. Another constraint is CBCT's limited contrast resolution for soft tissues [15]. As a result, it is inadequate for evaluating soft tissue lesions or structures such as muscles, lymph nodes, or the TMJ disc. Finally, while CBCT technology is becoming increasingly common, it represents a significant financial investment, which may restrict access in certain clinical settings, particularly in low‐resource environments.

Given the potential biological risks associated with X‐rays, numerous professional organisations have established guidelines to ensure the safe and appropriate use of CBCT in dental practice. The foundational principle of “As Low As Reasonably Achievable” (ALARA) underscores the necessity to minimise radiation exposure while still achieving the necessary diagnostic objectives. Building upon this, the concept of “As Low As Diagnostically Acceptable” (ALADA) was introduced to emphasise the balance between dose reduction and maintaining diagnostic efficacy. More recently, this has evolved into “As Low As Diagnostically Acceptable being Indication‐oriented and Patient‐specific” (ALADAIP), which advocates for tailoring imaging decisions not only to the diagnostic task but also to individual patient characteristics, thereby promoting more cautious and personalised use of CBCT in clinical practice [37].

While the precise level of risk associated with diagnostic imaging remains a matter of ongoing scientific discussion, substantial evidence demonstrates that exposures to ionising radiation elevate the likelihood of cancer manifestation over time. Notably, in an Australian cohort of 10.9 million individuals from infancy through late adolescence, CT exposure was associated with a 24% increase in cancers (brain tumours and leukaemia) with risk strongly correlated with higher dose and younger age during exposure [38].

Although the individual risk from dentomaxillofacial imaging is relatively small, the large number of patients exposed to diagnostic imaging renders this a significant public health concern. Reported effective doses from CBCT examinations exhibit substantial variability depending on the imaging protocol and field of view. In adults, doses range from 46 to 1073 μSv for large FOVs, 9–560 μSv for medium FOVs, and 5–652 μSv for small FOVs. Paediatric doses are similarly variable, ranging from 13 to 769 μSv for large or medium FOVs and 7–521 μSv for small FOVs. Mean effective doses in adults were 212 μSv for large FOVs, 177 μSv for medium FOVs, and 84 μSv for small FOVs, whereas in children, the corresponding mean doses were 175 μSv for large/medium FOVs and 103 μSv for small FOVs. Analysis of previous cohorts supports an increased risk of cancer from acute exposures in the 10–50 mSv range and chronic exposures in the 50–100 mSv range [33].

In this context, CBCT should be employed only when lower‐radiation alternatives, such as conventional radiography, are insufficient to provide the necessary diagnostic information. Exposure parameters should be tailored to the specific diagnostic task, with selection of the smallest FOV and lowest exposure settings compatible with acceptable image quality [17, 18]. Clinicians must possess adequate training in CBCT image acquisition, interpretation, and radiation protection to ensure safe and effective use. Major dental and radiological societies, including the American Academy of Oral and Maxillofacial Radiology (AAOMR) [39, 40], the International Congress of Oral Implantologists (ICOI) [41], the European Association for Osseointegration (EAO) [42], the American Academy of Periodontology (AAP) [43], the American Association of Endodontists (AAE) [39], the European Society of Endodontology (ESE) [44], and the European DIMITRA Project [45], have published consensus statements and position papers to guide the responsible implementation of CBCT in clinical practice. These guidelines, summarised in Table 1, delineate the specific clinical scenarios in which CBCT is indicated across different dental specialties, such as implantology, periodontology, endodontics, orthodontics, and paediatric dentistry, while consistently emphasising adherence to the ALARA, ALADA, and ALADAIP principles.

**TABLE 1:** Cone beam computed tomography (CBCT) indications across dental specialties according to professional guidelines.

Dental field	Indication for CBCT use	Source
Endodontics	When contradictory or non‐specific clinical signs and symptoms are present in untreated or previously treated teeth, and additional three‐dimensional information may improve diagnosis, treatment planning, or clinical management. A limited field of view should be considered the modality of choice.	American Association of Endodontists (AAE) and American Academy of Oral and Maxillofacial Radiology (AAOMR)—Joint Position Statement; European Society of Endodontology (ESE)
Implantology	When the implant receptor or bone augmentation sites are suspected, and conventional radiography cannot adequately assess the three‐dimensional anatomy. The smallest possible field of view should be used, and the entire image volume should be interpreted.	International Congress of Oral Implantologists (ICOI) and European Association for Osseointegration (EAO)
Orthodontics	When planning orthognathic surgery or correcting jaw asymmetry, supporting the safe and justified use of CBCT in dentistry.	American Academy of Oral and Maxillofacial Radiology (AAOMR)
Paediatric Dentistry	When evaluating orofacial clefts and planning tooth autotransplantation procedures.	European DIMITRA Project
Periodontology	When additional three‐dimensional information can enhance clinical decision‐making and treatment planning. Routine use of CBCT for diagnosing and treating moderate‐to‐severe periodontitis is not currently justified.	American Academy of Periodontology (AAP)

### 3.4 Technological Innovations in CBCT

Ongoing research and development efforts aim to further refine CBCT technology, enhancing image quality while reducing radiation exposure. Advances in detector sensitivity and image reconstruction algorithms have enabled the development of low‐dose CBCT protocols suitable for specific diagnostic applications; however, it should be noted that testing and adherence to optimised low‐dose protocols remain limited, particularly in paediatric patients [46]. Moreover, emerging software‐based solutions employ sophisticated algorithms to mitigate the impact of artefacts caused by metallic objects. Notably, metal artefact reduction (MAR) algorithms can improve diagnostic accuracy in cases involving restorations, implants, or endodontic materials [47, 48, 49]. However, it is important to acknowledge that their effectiveness may be limited for specific diagnostic tasks [50, 51] and in circumstances where metal is located in the exomass—the region surrounding the field of view (FOV) that does not appear in the image but interacts with the primary X‐ray beam, thereby producing metal‐related artefacts [1, 52, 53].

In parallel, AI and machine learning technologies are being increasingly incorporated into CBCT imaging workflows. These developments include automated segmentation of anatomical structures, facilitating detection of pathological findings, and contributing to the enhancement of image quality through noise reduction and artefact correction [54, 55, 56, 57, 58]. Collectively, such innovations hold the potential to streamline interpretation, reduce clinician workload, and improve diagnostic consistency.

Moreover, the integration of CBCT with other digital technologies has further expanded its clinical utility. The fusion of CBCT datasets with intraoral optical scans enables the creation of comprehensive virtual patient models, supporting advanced applications. These include precise virtual planning for guided implant surgery and the fabrication of customised surgical guides, preoperative planning and simulation of outcomes in orthognathic and reconstructive surgery, as well as the design of patient‐specific prosthetics and grafts [59, 60, 61]. Additionally, CBCT's capacity to provide reproducible 3D datasets enhances its value in forensic dentistry, particularly in applications related to personal identification and age estimation [56, 62, 63, 85].

## 4 Emerging Trends in 3D Imaging

The trajectory of dental radiology is increasingly shaped by technological innovations that extend beyond 2D and even current three‐dimensional (3D) imaging capabilities. These developments are not only enhancing diagnostic precision but are also reshaping clinical workflows and patient care paradigms. Key emerging trends include the integration of AI into image acquisition and interpretation, the evolution of digital workflows, and the expansion of non‐ionising imaging modalities.

**FIGURE 8:**
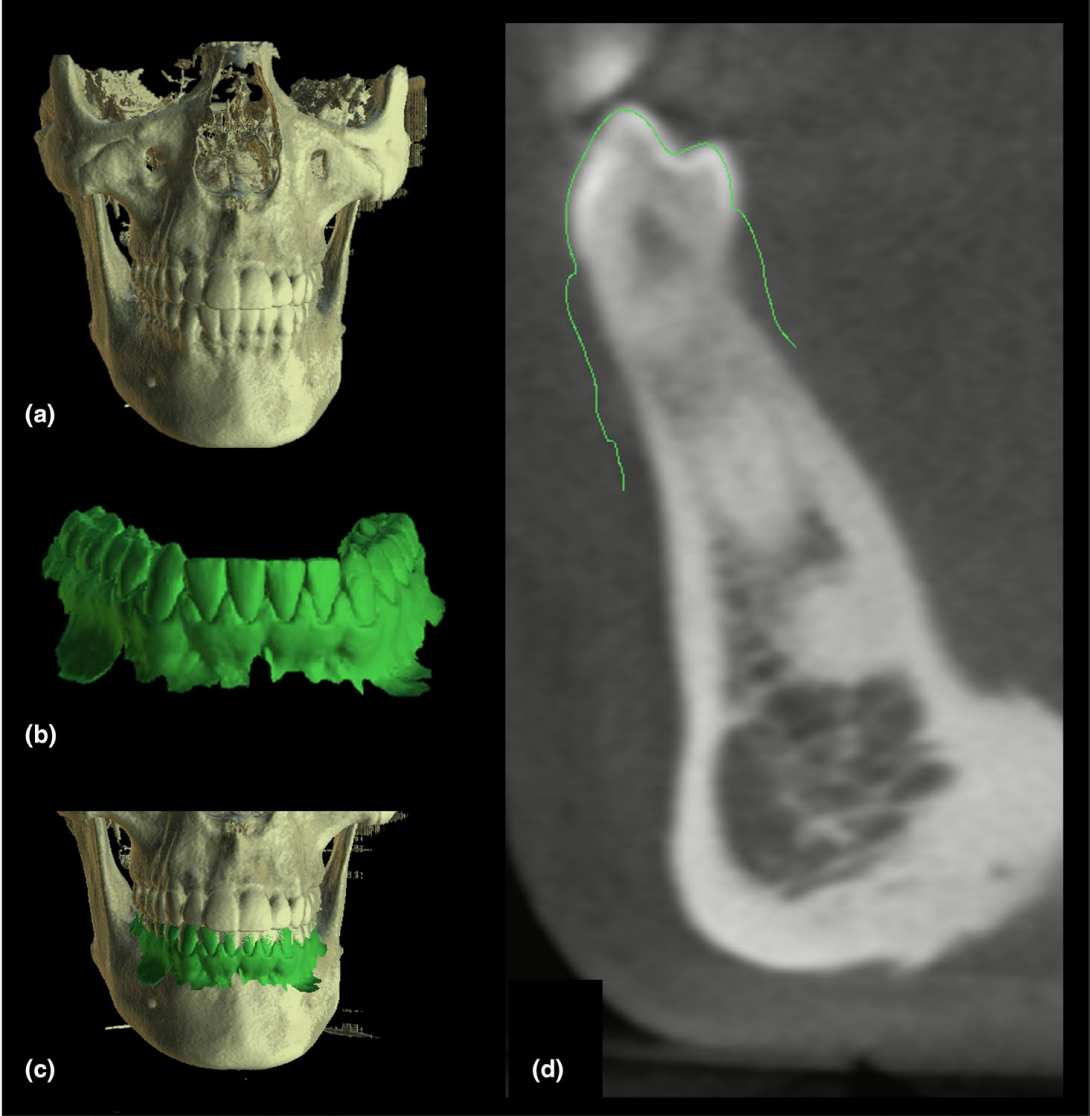
Integration of cone‐beam computed tomography (CBCT) (a) and intraoral scanning (b) for delineation of buccal gingival contours. The fused dataset is shown in (c), while the cross‐sectional view in (d) highlights the buccal gingival contours, indicated by the green line.

AI, particularly deep learning algorithms, is making significant advancements in dental imaging [13, 55]. Automated detection and classification of anatomical landmarks, dental pathologies such as caries, periapical lesions, and periodontal bone loss, are becoming increasingly accurate and reliable [54, 64, 65]. AI‐powered tools facilitate the interpretation of complex datasets, notably those generated by CBCT, reducing diagnostic time while enhancing reproducibility and accuracy (Figure 8). Moreover, AI‐driven image enhancement techniques enable noise reduction and artefact minimization, thereby allowing for lower radiation dose protocols without compromising diagnostic utility [56, 66]. The integration of these systems into clinical practice promises to standardise diagnostic processes, reduce interobserver variability, and improve patient outcomes.

**FIGURE 9:**
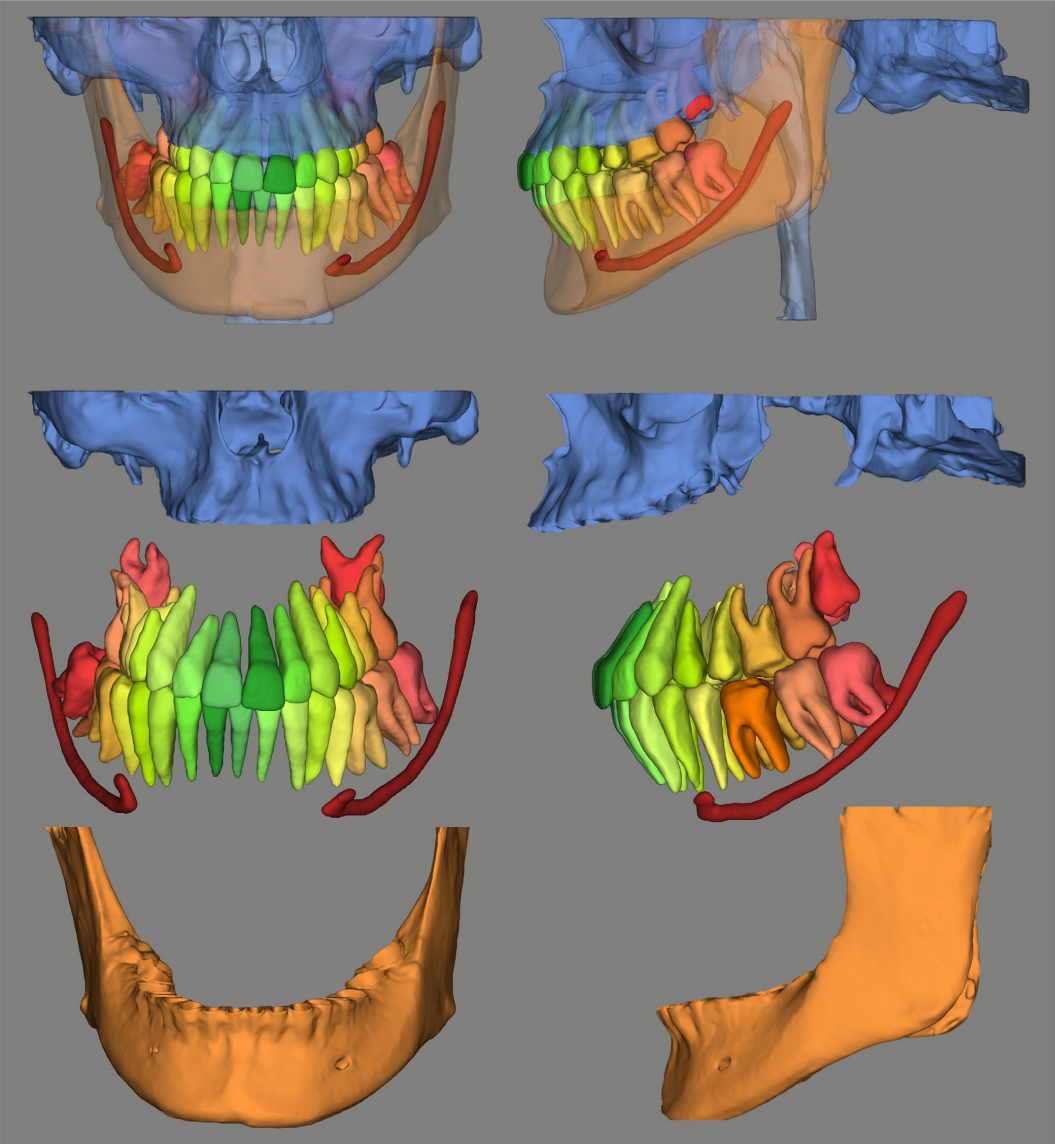
Segmentation of dentomaxillofacial structures from cone‐beam computed tomography data using the Relu online platform, presented as frontal and lateral views in both full and exploded views.

The digitization of dental practice extends beyond imaging into the entire clinical workflow. Digital impressions, CAD/CAM systems, and 3D printing technologies are now integrated with imaging data, enabling streamlined planning and execution of procedures. For example, data from intraoral scanners can be combined with CBCT datasets to fabricate surgical guides for implant placement, design orthodontic appliances, and facilitate the evaluation of buccal gingival contours, thereby enhancing both functional and aesthetic treatment outcomes (Figure 9) [32, 60]. Furthermore, cloud‐based platforms facilitate remote consultations, interdisciplinary collaboration, and data sharing, fostering a more connected and efficient healthcare environment. These developments contribute to patient‐centered care, enhancing communication, treatment planning, and overall satisfaction.

Growing concerns regarding cumulative radiation exposure, particularly in young patients, are encouraging research into non‐ionising imaging modalities such as magnetic resonance imaging (MRI) and ultrasound [28, 67, 68, 69, 70]. These technologies offer the potential to visualise soft and, increasingly, hard tissues without ionising radiation, aligning with radiation protection principles. A recent study evaluating health and pathology of periodontal and peri‐implant tissues revealed that MRI can visualise soft‐tissue inflammation, bone edema, and periodontal bone loss, but clinical evidence for peri‐implant tissue assessment is scarce; additionally, MRI is costly, time‐consuming, and requires substantial space compared to CBCT or intraoral radiographs. In addition, ultrasound can reliably assess gingival height, crestal bone level, and furcation involvement, although its use is generally limited to buccal surfaces due to physical constraints [71]. Infrared thermography, for instance, has been tested for the assessment of patients with temporomandibular disorders, enabling non‐invasive evaluation of thermal patterns associated with inflammation and dysfunction; however, the findings to date have been inconclusive and have not consistently demonstrated robust diagnostic value [72]. Conversely, optical imaging modalities, including optical coherence tomography, have demonstrated satisfactory results in periodontal and peri‐implant assessments [73, 74], but their utility remains largely preclinical, and it may fail to reach the base of inflamed pockets due to light absorption by haemoglobin [71].

Another emerging trend is the convergence of different imaging modalities. Multimodal imaging combines the strengths of various techniques, such as the high spatial resolution of CBCT with the superior soft tissue contrast of MRI, enabling comprehensive assessments of complex clinical cases [75]. Software platforms capable of fusing datasets from different sources allow clinicians to simulate surgical interventions, perform precise measurements, and visualise anatomical relationships in three dimensions. This integrative approach is particularly valuable in diagnostic tasks where detailed anatomical knowledge is key.

## 5 Non‐Ionising Imaging Modalities: Expanding the Horizons of Dental Diagnostics

The exploration of non‐ionising imaging modalities in dentistry is gaining attention in recent days as part of a broader commitment to minimise patient exposure to ionising radiation while expanding diagnostic capabilities. Among the most promising technologies in this regard are MRI and ultrasound. These modalities offer unique advantages, particularly in soft tissue imaging, and are increasingly being adapted to overcome traditional barriers to their widespread adoption in dental practice.

### 5.1 Magnetic Resonance Imaging (MRI)

MRI operates by utilising the magnetic properties of hydrogen atoms in the body to generate detailed images of soft tissues, without using ionising radiation. Its superior contrast resolution makes it the modality of choice in many medical fields for evaluating soft tissue structures [7]. In dentistry, MRI's potential has long been recognised for imaging the TMJ, salivary glands, and pathological lesions of soft tissues. Key advantages of MRI include its non‐invasive nature, absence of ionising radiation, and its capability to provide multiplanar imaging. Furthermore, MRI is sensitive to changes in tissue composition, making it useful for detecting inflammatory changes, neoplasms, or cystic lesions [67, 76].

Historically, MRI's application in dentistry has been limited by its poor visualisation of mineralized tissues such as bone and teeth, as well as by cost and accessibility barriers. However, recent technological advances are addressing these limitations, with MRI demonstrating successful application in the assessment of peri‐implant bone lesions [77]. Moreover, the advent of high‐field MRI systems (e.g., 3 T and above) has enhanced image resolution and signal‐to‐noise ratios, providing more detailed anatomical information. Kinetic MRI is also being explored for the assessment of the mandibular condyle in patients with TMJ disorders [78].

**FIGURE 10:**
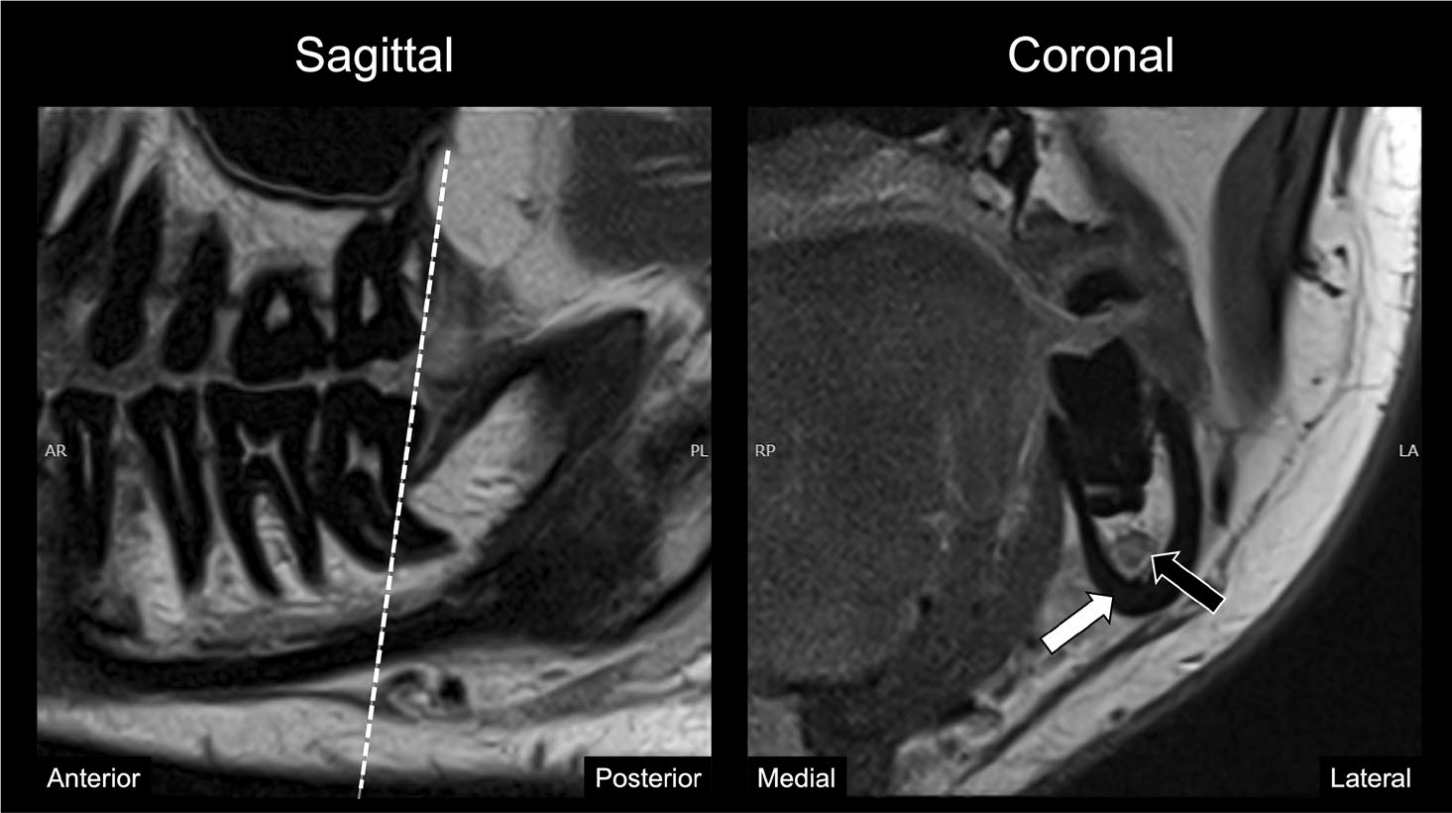
Sagittal and coronal proton‐density‐weighted magnetic resonance images (Siemens MAGNETOM Free. Max 0.55 T; Siemens Healthineers, Forchheim, Germany) acquired with a 7‐channel radiofrequency dental‐dedicated receive coil (RAPID Biomedical GmbH, Rimpar, Germany; resolution: 0.3 × 0.3 × 2 mm; acquisition time: 2′38″; repetition time: 2400 ms; echo time: 48 ms; flip angle: 150°; bandwidth: 100 Hz/pixel; GRAPPA acceleration: × 3, averages: 4) demonstrating the posterior region of the jaws. The images reveal a hyposignal of the teeth and cortical bone (white arrow), as well as a soft tissue‐like signal associated with the dental pulp and mandibular canal (black arrow). The dashed line delineates the anatomical plane from which the coronal section was acquired. Courtesy of Prof. Dr. Rubens Spin‐Neto, Aarhus University, Denmark.

**FIGURE 11:**
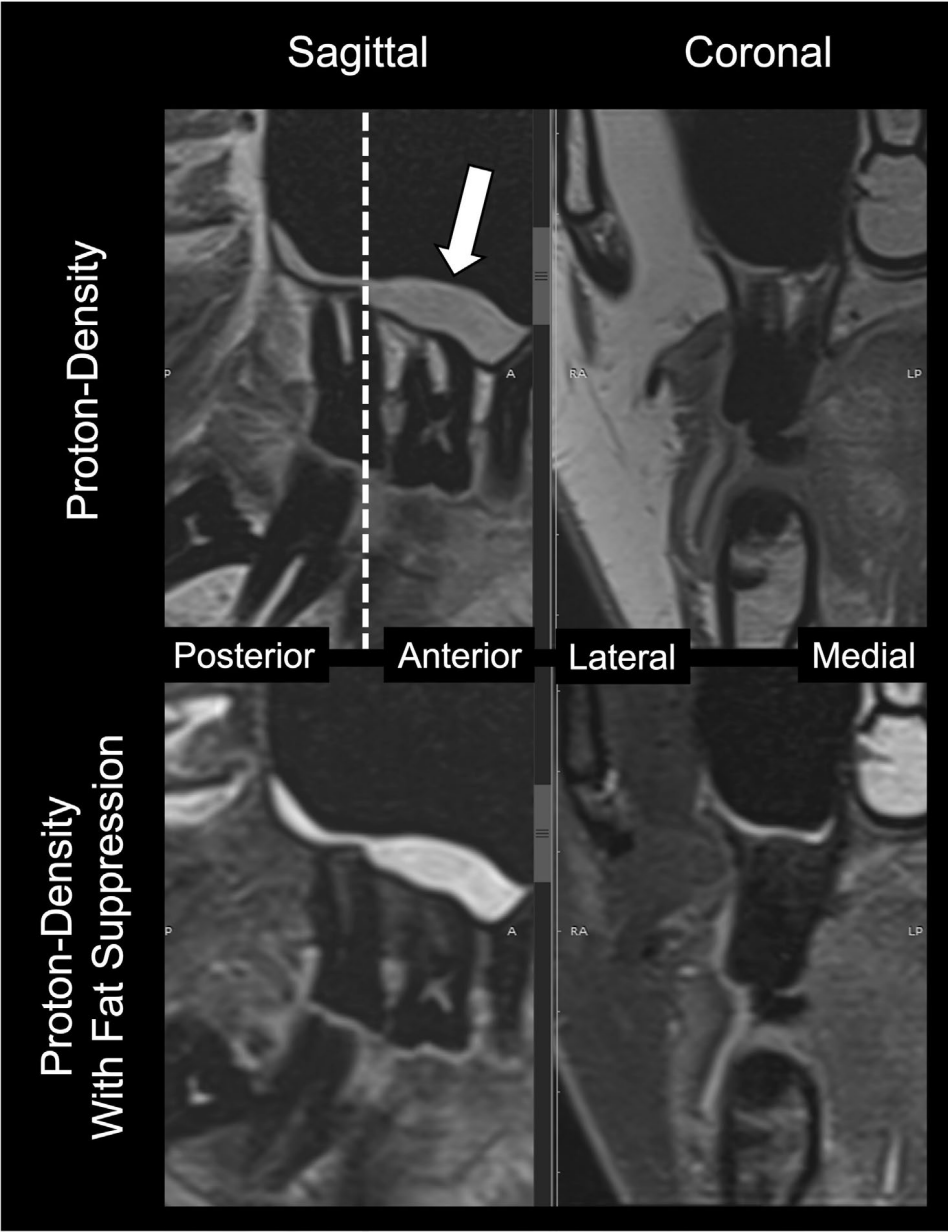
Sagittal and coronal proton‐density‐weighted magnetic resonance images (Siemens MAGNETOM Free. Max 0.55 T; Siemens Healthineers, Forchheim, Germany) acquired with a 7‐channel radiofrequency dental‐dedicated receive coil (RAPID Biomedical GmbH, Rimpar, Germany; resolution: 0.3 × 0.3 × 2 mm; acquisition time: 2′38″; repetition time: 2400 ms; echo time: 48 ms; flip angle: 150°; bandwidth: 100 Hz/pixel; GRAPPA acceleration: × 3, averages: 4). The posterior region of the jaws revealing sinus mucosal thickening (white arrow), more evident with fat suppression (Short Tau Inversion Recovery—STIR; resolution: 0.4 × 0.4 × 2 mm, acquisition time: 3′08″; repetition time 2560 ms; echo time 46 ms; flip angle: 130°; bandwidth: 106 Hz/pixel; GRAPPA acceleration: × 3; averages: 6, STIR). The dashed line indicates the anatomical plane from which the coronal section was acquired. Courtesy of Prof. Dr. Rubens Spin‐Neto, Aarhus University, Denmark.

More recently, dental‐dedicated MRI (ddMRI) introduced an innovative, MRI method tailored for precise evaluation of structures within the dentomaxillofacial region. Unlike traditional MRI protocols, which typically employ high‐field systems (1.5–3 T) in general medical environments, ddMRI utilises a lower magnetic field strength of 0.55 T. The system is specifically engineered with optimised radiofrequency surface coils for enhanced signal detection in the oral and maxillofacial region, while ensuring patient comfort. Moreover, specialised pulse sequences and modern image reconstruction strategies, such as parallel imaging and machine learning‐based algorithms, allow for high‐quality diagnostic images to be obtained rapidly, typically within 3 min (Figures 10 and 11) [69]. Low‐field imaging also brings technical advantages, including diminished artefacts caused by metal‐based dental materials and reduced signal distortion near air‐tissue boundaries. Collectively, these innovations address prior limitations of MRI in dentistry—such as lengthy scan times, susceptibility to artefacts, and infrastructural constraints—while offering new opportunities for early diagnosis, disease prevention, and potentially more autonomous imaging by dental practitioners. To date, ddMRI has shown promising outcomes in the follow‐up of lower third molar removal and comparable performance to a 1.5 T MRI system in the evaluation of the TMJ [20, 79]. However, ongoing investigations are necessary to establish ddMRI's clinical accuracy across its many possible applications.

Despite the numerous potential benefits of MRI, several major limitations persist: it remains a potential source of claustrophobia, is costly, and is less accessible in dental settings compared to conventional radiography or CBCT. Additionally, patient contraindications—such as the presence of ferromagnetic implants—may restrict its use.

Building on recent advances in MRI, AI is increasingly being applied to further enhance its diagnostic potential. AI‐driven approaches, particularly machine learning and deep learning algorithms, can automate segmentation of anatomical structures, detect subtle pathological changes, and quantify tissue characteristics that may not be easily appreciated by the human eye [80]. Radiomics, the extraction of high‐dimensional quantitative features from MRI images, allows for detailed characterisation of tissue heterogeneity, inflammation, and bone remodelling patterns. When combined with AI, radiomics can support predictive modelling, treatment planning, and longitudinal monitoring of dental and maxillofacial conditions [81], potentially transforming MRI from a primarily qualitative tool into a more objective and data‐driven modality in clinical dentistry.

### 5.2 Ultrasound

**FIGURE 12:**
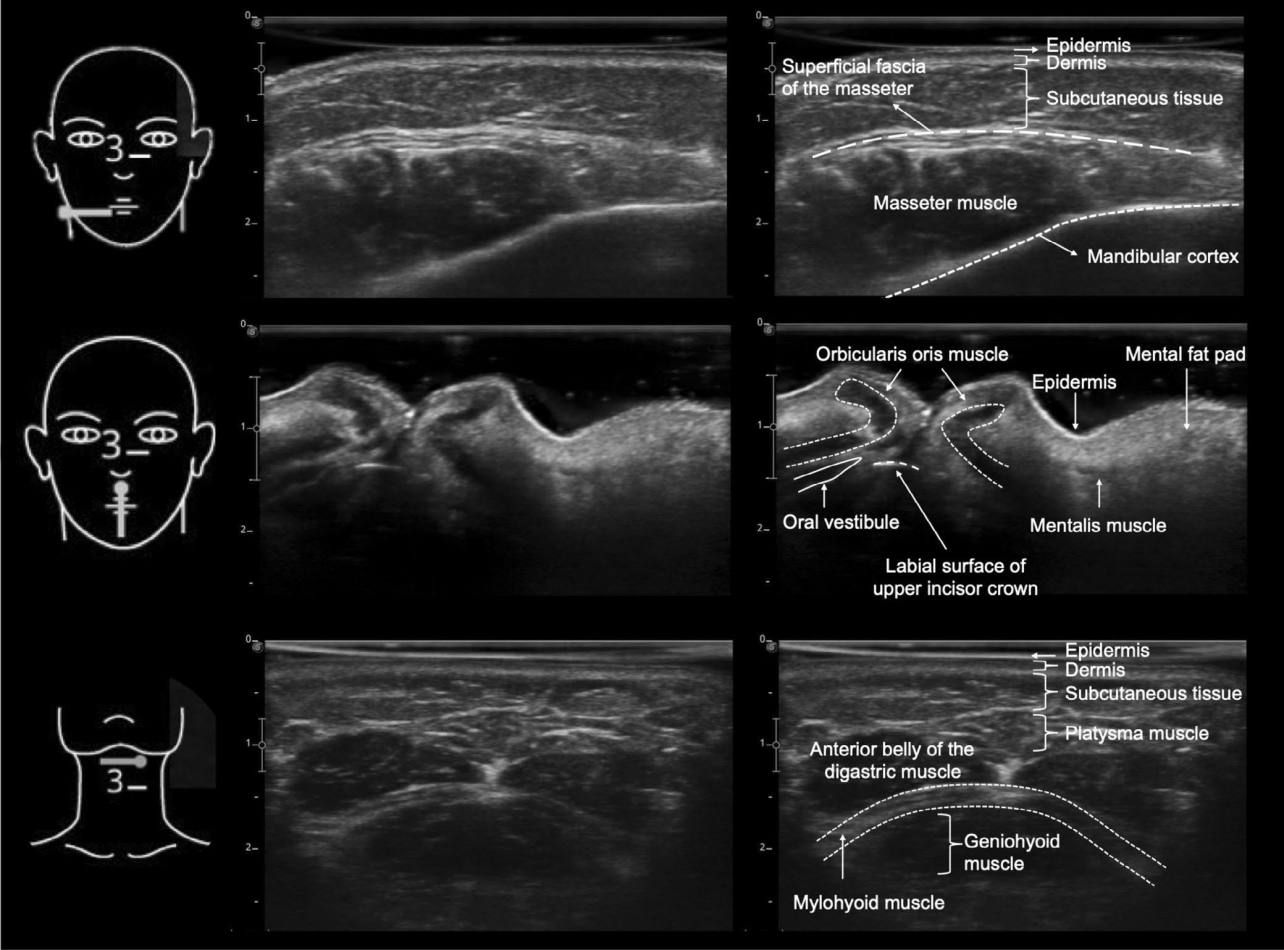
Ultrasonographic images with annotations of anatomic structures obtained with the probe positioned horizontally at the base of the mandible, vertically at the center of the lips, and horizontally in the submental region, respectively. All images were acquired using a portable EVUS 5 system (Alliage, Ribeirão Preto, Brazil) with a linear array transducer model L741 (Alliage, Ribeirão Preto, Brazil). For the base of the mandible and center of the lips, the transducer operated at a frequency range of 10–13.5 MHz, a beam depth of 3 cm, and a gain of 115, with focal zones of 0.5 and 1 cm, respectively. For the submental region, the transducer operated at a frequency range of 10.9–14.0 MHz, a beam depth of 3 cm, a gain of 135, and a focal zone of 0.5 cm. Courtesy of Dr. Carol Padilla, Brazil.

Ultrasound imaging, also referred to as echography, employs high‐frequency sound waves to generate real‐time images of soft tissues. It is inherently safe, portable, cost‐effective, and free from ionising radiation. These attributes make it an attractive adjunct for various dental diagnostic purposes. Ultrasound is well‐suited for evaluating superficial soft tissues and is particularly effective in imaging salivary glands, lymph nodes, vascular structures, and superficial masses (Figure 12). It is also widely used for guiding minimally invasive procedures, such as fine‐needle aspiration biopsies of salivary gland lesions [7, 68, 82].

Recent improvements in ultrasound technology, including higher‐frequency probes and enhanced image processing algorithms, have expanded its potential applications in dentistry. For example, intraoral ultrasound probes enable the evaluation of periodontal structures, including gingival thickness and the detection of periodontal pockets, with good correlation to conventional probing techniques [70]. Moreover, ultrasound has shown promise in the detection of periapical pathology, such as abscesses or cystic lesions, and in the assessment of TMJ components, particularly the evaluation of joint effusion and muscle abnormalities. Ultrasound is also being investigated for measuring alveolar bone levels and monitoring wound healing after surgical procedures [82]. Its dynamic, real‐time nature allows for the assessment of functional aspects, such as muscle movement and vascular flow, which are not easily evaluated with static imaging modalities.

Despite its advantages, ultrasound is highly operator‐dependent, requiring specific training to acquire and interpret diagnostic‐quality images accurately. Additionally, its inability to penetrate mineralized tissues significantly limits its utility in imaging intraosseous structures, such as the alveolar bone, teeth, and deep‐seated lesions. The presence of acoustic shadowing behind calcified structures can obscure relevant anatomical details, limiting its applicability for certain diagnostic tasks that remain to fall within the scope of X‐ray‐based techniques.

Unlike the other imaging modalities discussed, such as 2D radiography, CBCT, and MRI, ultrasound remains at an earlier stage of clinical integration in dentistry. Consequently, the application of AI to ultrasound imaging has yet to achieve meaningful progress in this field. This lag likely reflects the still‐limited adoption of ultrasound in dental practice, which restricts the generation of large, standardised datasets required for AI model training and validation.

### 5.3 Other Non‐Ionising Modalities

Emerging optical imaging technologies, such as optical coherence, are also being explored in dentistry in the evaluation of periodontal and peri‐implant conditions [73, 74]. While these modalities remain largely investigational, they highlight the expanding interest in non‐ionising diagnostic approaches.

## 6 Critical Appraisal: Where Are We Now?

The evolution from 2D radiography to advanced 3D imaging modalities has undeniably revolutionised dental diagnostics, offering unprecedented anatomical detail and improving clinical decision‐making across various specialties. However, this technological progress must be critically appraised in light of its associated risks, costs, and the necessity for careful application.

While CBCT inherently provides volumetric data and can deliver relatively high spatial resolution images of hard tissues, its widespread use raises concerns about radiation dose, particularly given the increasing frequency of imaging in routine dental care. The principle of justification remains paramount, necessitating that the benefits of each imaging procedure outweigh the potential risks. Additionally, image interpretation requires advanced training to avoid misdiagnosis, particularly in recognising artefacts and incidental findings.

Emerging trends, notably the integration of AI and the exploration of non‐ionising modalities, present promising opportunities to address some of these challenges. AI has the potential to enhance diagnostic accuracy, reduce clinician workload, and minimise radiation exposure through optimised imaging protocols. Similarly, non‐ionising imaging techniques such as MRI and ultrasound offer safer diagnostic options for specific clinical scenarios, although they currently function more as complementary tools than direct replacements.

Looking forward, the future of dental imaging lies in personalised, precision diagnostics characterised by integrated multimodal imaging, automated analysis, and patient‐specific risk assessment. The increasing interoperability of digital systems will further facilitate comprehensive treatment planning and interdisciplinary collaboration. Nonetheless, making this future a reality requires addressing several challenges such as making sure everyone has fair access to these advanced imaging technologies, standardising training and competency across the profession, and establishing robust regulatory frameworks to guide how AI should be used in patient care.

## Conflicts of Interest

The author declares no conflicts of interest.

## Data Availability

Data sharing not applicable to this article as no datasets were generated or analysed during the current study.
